# Alternative Ultrasound Gel for a Sustainable Ultrasound Program: Application of Human Centered Design

**DOI:** 10.1371/journal.pone.0134332

**Published:** 2015-08-07

**Authors:** Margaret Salmon, Christian Salmon, Alexa Bissinger, Mundenga Mutendi Muller, Alegnta Gebreyesus, Haimanot Geremew, Sarah Wendell, Aklilu Azaza, Maurice Salumu, Nerys Benfield

**Affiliations:** 1 University Health Network, University of Toronto, Department of Emergency Medicine, Toronto, Canada; 2 Western New England University, Department of Industrial Engineering and Engineering Management, Springfield, Massachusetts, United States of America; 3 University of California San Francisco, Department of Emergency Medicine, San Francisco, California, United States of America; 4 HEAL Africa Hospital, Department of Emergency Medicine, Goma, Republique Democratique du Congo; 5 Black Lion Hospital, Department of Emergency Medicine, Addis Ababa, Ethiopia; 6 Black Lion Hospital, Emergency Medicine Research Center, Addis Ababa, Ethiopia; 7 Georgetown University, School of Medicine, Washington, D.C., United States of America; 8 Addis Ababa University, Tikur Anbessa Specialized Hospital, Department of Emergency Medicine, Addis Ababa, Ethiopia; 9 Kindu General Hospital, Department of Internal Medicine, Kindu, Democratic Republic of Congo; 10 Albert Einstein College of Medicine of Yeshiva University, Department of Obstetrics & Gynecology, New York, New York, United States of America; San Raffaele Scientific Institute, ITALY

## Abstract

This paper describes design of a low cost, ultrasound gel from local products applying aspects of Human Centered Design methodology. A multidisciplinary team worked with clinicians who use ultrasound where commercial gel is cost prohibitive and scarce. The team followed the format outlined in the Ideo Took Kit. Research began by defining the challenge "how to create locally available alternative ultrasound gel for a low-resourced environment? The "End-Users," were identified as clinicians who use ultrasound in Democratic Republic of the Congo and Ethiopia. An expert group was identified and queried for possible alternatives to commercial gel. Responses included shampoo, oils, water and cornstarch. Cornstarch, while a reasonable solution, was either not available or too expensive. We then sought deeper knowledge of locally sources materials from local experts, market vendors, to develop a similar product. Suggested solutions gleaned from these interviews were collected and used to create ultrasound gel accounting for cost, image quality, manufacturing capability. Initial prototypes used cassava root flour from Great Lakes Region (DRC, Rwanda, Uganda, Tanzania) and West Africa, and bula from Ethiopia. Prototypes were tested in the field and resulting images evaluated by our user group. A final prototype was then selected. Cassava and bula at a 32 part water, 8 part flour and 4 part salt, heated, mixed then cooled was the product design of choice.

## Background

Clinician-performed point of care ultrasound (POCUS) is emerging as a useful diagnostic tool for healthcare providers in limited-resource settings. [1–2] Several early studies demonstrated that ultrasound significantly impacts diagnosis and management of patients. [3] The value of ultrasound has also gained increasing recognition by ministries of health in low and middle-income countries (LMICs), non-governmental organizations (NGOs), and the World Health Organization (WHO). [4]

Despite the evidence, utilization of bedside ultrasonography by providers in low-income settings remains limited. Lack of portable ultrasound machines is certainly a primary reason. Other common implementation barriers include lack of adequate training and limited access to consumable supplies required, such as ultrasound gel. [5–7] Indeed, the cost and availability of commercially produced ultrasound gel in rural, limited resourced healthcare settings is prohibitive and limits the number and quality of scans available to clinicians even with in-place equipment.

This situation is unfortunate, as persons in lower resource countries have higher morbidity and mortality in obstetric and trauma care, two of the areas in which ultrasound has proved most useful for patient management. [4,8] This disparity also raises significant global health equity concerns: equal access to healthcare and health technology is an important part of the worldwide strategy to reduce global disparities in health and achieve the health-related millennium development goals.

Alternative gels have been suggested in previous research. The “WHO-*Manual of Diagnostic Ultrasound*” suggests a mixture of materials involving a source-intensive industrial process not readily available in most rural settings. [9] Cornstarch based mixtures have also been successfully tested. [5] Unfortunatly, cornstarch is not readily available or produced in many geographically isolated settings, leaving cost and availability restrictions intact.

Research presented here describes the design of potential gel alternatives that employs locally available materials (roots, grains and other food stuffs) identified via Human Centered Design (HCD), a qualitative rapid solution-generating design methodology to approach open-ended problems. For ease of project completion the design team used the open-source HCD IDEO Took Kit. (IDEO Tool Kit, 2011) funded by Bill & Melinda Gates Foundation, as a guide. [10] We chose HCD methodology specifically in order to capitalize on local knowledge, allow a search for solutions from unexpected sources and to provide an opportunity for rapid iterations for quick solutions. The process has previously led to innovations such as the HeartStart defibrillator, CleanWell natural antibacterial products and has been used in business product innovation for many years. [10]

Despite an extensive Pub Med search, to the best of our knowledge this is one of few applications of HCD methodology described in the medical literature to generate a new clinical product that explicitly addresses scarcity and cost by transferring the sourcing and production of consumable materials to the local economy. Importantly, we highlight this application of HCD methodology to suggest how it might be applied in the future to address other clinical consumable shortages.

## Methods

### Concept of Human Centered Design

Human Centered Design (HCD), also called user-centered design or design thinking, is a solution-design process of user empathy, purposeful design, rapid successive prototyping and iteration, integrating user feedback to generate feasible, desirable, and practical solutions. The IDEO HCD Tool Kit is a process guide focused on creating solutions for low-resource settings. The guide outlines the stages of the HCD process and suggests potential methodology and exercise for these settings. This specific guide divides the design process into three phases:

1. “**H**ear:” Understand and empathize with end-users using participant observation, site visits, and ethnographic interviewing. These materials are then used to define a design challenge.
2. “**C**reate:” Apply brainstorm techniques, visual layout and needs statement synthesis to guide rapid prototyping to elicit feedback from end-users.
3. “**D**eliver:” Provide prototypes to end-users to react to and incorporate their feedback into the next cycle of iteration.

### Identification of Design Challenge

#### Design Team

Our design team was made up of a diverse group of six individuals from different professional and national backgrounds: an MD-MPH emergency medicine physician and ultrasound expert, Canada (F); an MD, emergency medicine house staff and ultrasound user, from Democratic Republic of Congo (M); a civil engineer, from USA (M); a filmmaker, Democratic Republic of Congo (M); a business student, Democratic Republic of Congo; a physician and entrepreneur, DRC (M) in order to bring multiple perspectives to the table to diversify solution generating perspectives. An additional HCD trained emergency medicine physician MD MBA, USA (F) was available to advise on the HCD process.

Based on prior interviews and in-field experience, we defined our design challenge as:

How might we create an alternative ultrasound gel that would be locally available and affordable, that will yield a functional alternative to commercially produced gel for use in ultrasonography in low-resourced African environments?

### Study Participants

#### End-Users and Sites

We defined our End-Users as local clinicians who use point of care ultrasound (POCUS) from sites representative of low-resource clinical environments with broad geographic distribution for Sub-Saharan & Horn of Africa. Clinical field visits and interviews took place in the departments of obstetrics and emergency medicine at Heal Africa Hospital, trauma center Goma, Eastern DRC; Kindu General Hospital, Ministry of Health regional hospital, Maniama Province, DRC; and Black Lion/Tikur Anbessa Specialized Hospital, Department of Emergency Medicine, Addis Ababa, Ethiopia. Forty-nine healthcare providers participated. Thirteen staff emergency physicians in Ethiopia, twenty-six resident staff physicians and orthopedic officers in DRC and ten staff physicians in Kindu, DRC contributed their ideas and feedback to this project, over the course of several site visits.

#### Background Knowledge Experts

Background knowledge experts of POCUS ultrasound were identified as members of the ultrasound section of American College of Emergency Physicians (ACEP). ACEP is one of the most recognized professional emergency medicine organizations and the United States is a world leader in POCUS application.

#### Local Experts

Local supply and production experts were identified as local market vendors because of their in-depth knowledge of locally available starches and food products. Market vendors were recruited at Virunga Market, Goma DRC, City Centre, Kindu, DRC; Merkato in Lideta Subcity and Addis Ketema, Addis Ababa Ethiopia, and Banankabougou and Sébénikoro Quarter Market, Bameko Mali. While there were no End-Users in Mali, we felt it was important to include this market in order to ensure solutions include the region of West Africa. Only vendors who sold bulk starch-like grains and flours were recruited.

#### Reviewers

Two emergency medicine physicians with specialty training in ultrasound and a nurse radiologist with ultrasound training.

### Data Collection Methods

All information from brainstorming sessions and interviews were recorded as written notes in either English or French. The notes were used as working documents and no further transcriptions were done. Note takers were design group members during the brainstorm sessions and research assistants during the interviews.

#### Hear

The Design Team conducted field visits for one month in Goma (DRC) and Addis Ababa (Ethiopia) each and nine days in Kindu (DRC) to observe End-Users in the clinical setting. Both participatory observation and group interviews were conducted with the objective of obtaining existing knowledge of limitations to local POCUS use and challenges to using commercial ultrasound gel. The team then identified a group of Background Knowledge Experts and requested suggestions for alternatives to commercial gel for use in low-resource settings. The request was sent via email by ACEP organizers.

#### Create

A total of thirteen brainstorm sessions were held in the field. The first three were dedicated to describing product metrics. Metrics were gleaned from previous literature, (5) commercial ultrasound gel websites and conversations with End-Users. Brainstorming sessions were held in a designated meeting space with all Design Team members. A project leader recorded ideas and suggestions by written text. Ideas were discussed and if rejected through consensus crossed off the list, if approved they remained active until the next session. A laboratory was simultaneously established with hot plate, cook stove, ultrasound machine, vascular and abdominal probe, bottle, & ingredients (suggested products from the market, salt, water, spoons, pot) for prototype development. A designated project engineer prepared all prototypes recording ingredient combinations in a notebook. If a prototype was deemed acceptable by Design Team members it was presented to End-Users. If not, a line was crossed through the formula. Before any 3^rd^ party was exposed to any solution, Design Team members were recruited as proxies for prototype testing and scans for the project were done only on Design Team and End-Users.

**Fig 1:**
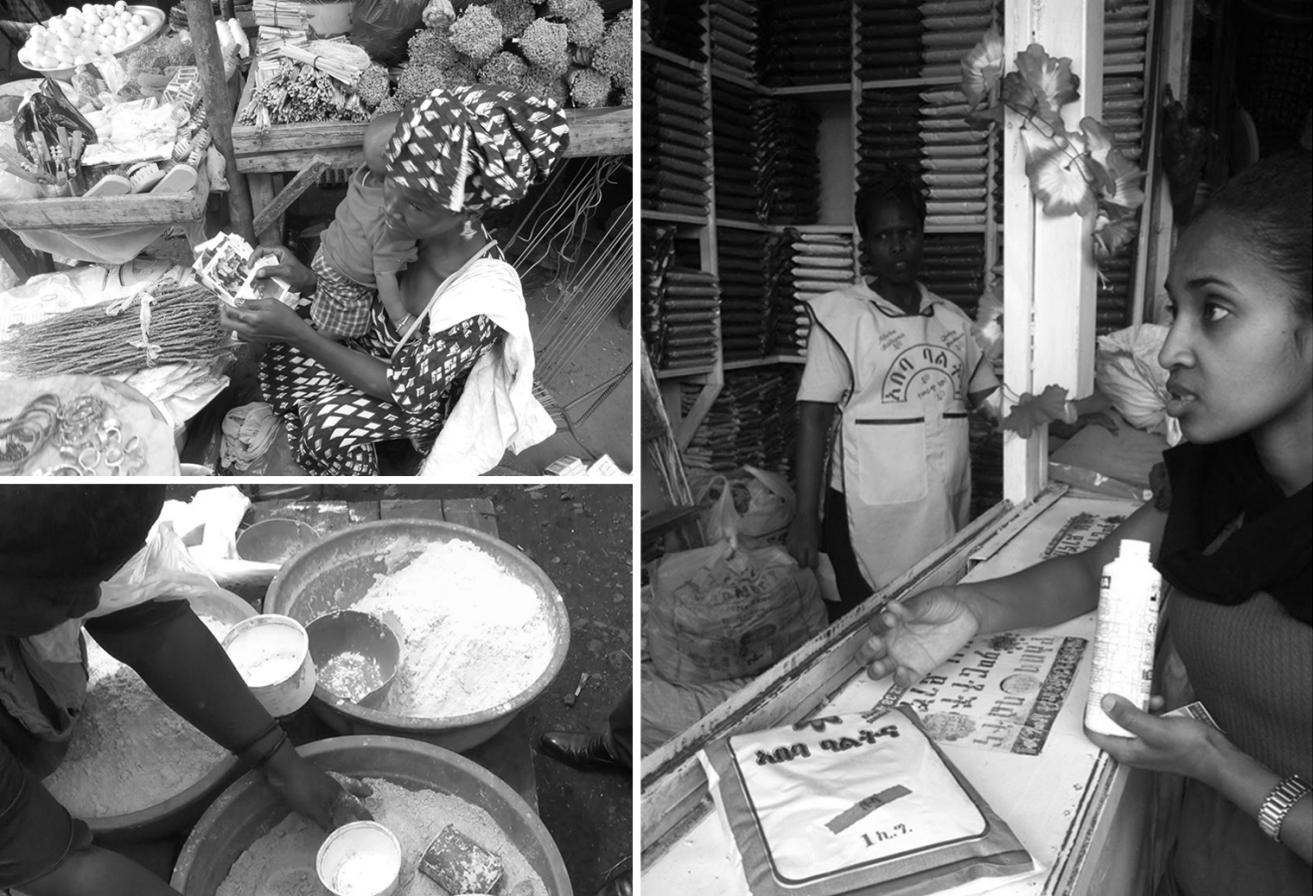
Markets in Goma, Addis Ababa, and Bameko. (A) Vendors are interviewed in Addis Ababa Ethiopia at Merkato in Lideta sub city and Addis Ketema by research colleague Alegnta Gebreyesus, MD in Amharik. (B) Vendors are interviewed at the Virunga Market in Goma, DRC by research assistant, Rene Zaidi, in Kiswahili. Commercial ultrasound gel is demonstrated and this vendor suggests banana flour, sorgum or cassava root flour. (C) Vendors are interviewed at Bameko Mali by research assistant, Bouba, in Bambera. The vendor reviews photos of obstetric ultrasounds and suggests banana flour, sorgum or cassava root flour.

Results of the online survey from Background Knowledge Experts were then queried for ready commercial gel alternatives that met those metrics and potential materials selected. Local Experts were then identified based on these results and interviewed by research assistants to query for local suggestions that were similar to the materials selected. 72 vendors in Kindu & Goma, Democratic Republic of Congo, Addis Ababa Ethiopia and Bameko Mali were then interviewed in groups. The women were shown photos of physicians performing ultrasonography and given samples of commercial gel and the design challenge was discussed. (Fig 1) Participation was determined if a vendor commented either to the research assistant or other vendors to discuss the topic. Interviews were done at the market place with suggestions collected in written format, and the interaction in still photo and video format.

#### Deliver

Local Expert interviews were reviewed and a prototype was developed from the suggestions. The prototype was first tested on Design Team members and then over the course of 10 days, presented to End-Users for feedback through discussion using the desired product metrics as a guide but with no particular objective scoring system. End-Users discussed metrics and each cycle of iteration incorporated the feedback from the previous cycle.

Once End-Users decided a prototype was sufficient, Reviewers evaluated the images. Reviewers were blinded and evaluated eight paired comparison images taken with prototype and commercial gel that had been recorded and downloaded to a laptop. Images were the 4 FAST views, hepato-renal, splenic renal, subxiphoid cardiac and pelvis FAST, median nerve block images and lung sliding video. No scoring system was incorporated into the review rather a qualitative assessment was made if the image was of equal quality as the images with commercial gel showing the organs or structure described in the International Federation of Emergency Medicine (IFEM) Point-of-Care Ultrasound Curriculum Guidance. [11] Costs per 250ml for manufacture were also calculated.

### Ethics Statement / Internal Review Board and Consent

Informed consent was obtained from all participants as outlined in PLOS consent form including End-Users and Local Experts. Consents for market vendors were verbal in either Swahil or French (DRC), Amharik (Ethiopia) or Bambara (Mali) by native speakers. Written consent was not obtained from market vendors given this population does not usually read or write and it was thought disingenuous to sign a form that they were not able to understand in a culture where signatures are normally not required. Market vendors whose image was captured by still or video photography however were consented in written form but only after extensive counseling. This process involved individual counseling regarding the project need for visual descriptors and an explanation that photos maybe in an open-source journal. The vendors were also shown the photo on the camera screen and given the opportunity to have them deleted. Research assistants requested a short return conversation from the vendor verbalizing their understanding regarding use of photos before consent was formalized. The IRB committee of Kindu General Hospital was aware of this consent process. Research assistants were provided study protocol training. This study was approved by the Internal Review Board of Kindu General Hospital, DRC. The research is presented in accordance with the consolidated criteria for reporting qualitative research (COREQ) checklist. [12]

## Results

### Hear

End-Users consistently described lack of ultrasound gel due to both cost and local access as a major limitation during field visits. When commercial ultrasound gel was used it was often is small quantities that image quality was compromised (HEAL Africa Hospital, Black Lion Hospital). Design Team members also witnessed local physicians attempting to utilize ultrasound with low-viscosity oils and lubricating gels with poor image results (Kindu General Hospital). End-Users at Black Lion Hospital in Ethiopia performed twice as many scans (30/day) as either site in DRC (5–10/day).

Twenty responses were received from Background Knowledge Experts with nine potential alternatives suggested. Alternative One: Water, which the Design Team determined not of sufficient viscosity for clear quality imaging. Alternative Two: Shampoo, determined more expensive than gel, Alternative Three: Guar Gum, unavailable for purchase in local markets of Kindu and Goma; Alternative Four: cornstarch slurry, determined too expensive. Alternative Five: gelatin, not available, Alternative Six: lotion, determined too expensive, Alternative Seven: Betadine, determined too expensive and could possibly damage the ultrasound probe. Alternative Eight: surgical lube, determined too expensive. Suggestions were narrowed down to best chance ingredient based on determinates of cost and availability.

Cornstarch was the most likely solution, given the abundance of corn in central and eastern DRC and Addis Ababa and previous validation for use. Cornstarch availability in the local market, however, ranged from limited (Addis Ababa) to non-existent (rural DRC). Further, research suggested that its production is a resource intensive industrial process to extract the germ from the grain before grinding. These considerations negated cornstarch as a viable solution for End Users.

### Create

Product metrics chosen were consistency/viscosity: equivalent to commercial gel by sight and feel. Durability: Maintain consistency while unrefrigerated with no separation of materials for > 48 hours. Practicality: Manufactured using locally available equipment, including electric heating element or open charcoal flame, in a time duration that can be reasonable performed on a daily basis. Cost: 1/100^th^ cost of commercial gel as relative to cost on local market (arbitrarily selected). Image acceptability: Reviewers noted no visual differences in image quality in-side by-side comparison with conventional gel.

Early in the brainstorming process, both cornstarch and other cooking products and foodstuffs came up as products with analogous physical properties to ultrasound gels. Given the familiarity of market vendors with local food products and their preparation, they were selected as an Expert Group (focus group) for interviewing and identifying possible local materials that could be prepared as ultrasound gel alternatives.

During group interviews (minimum two visits each market), market vendor suggestions included:

- Kindu and Goma DRC vendors: sorgum, mixture of egg/lemon/cassava, aloevera, plantain flour, cassava root flour, and hand sanitizer
- Addis Ababa, Ethiopia vendors: selit flour (sesame), telba, bula (flour from ensset), ajji, gondare and ret.
- Bameko Mali: suggested: Cassava, sorgum, banada flour and ripe fruit from the Mali Yurni tree.

### Deliver

Plaintain & sorgum flour, cassava root flour, bula and selit were the only suggestions which when mixed with water and heated formed a consistency similar to commercial gel. On day one, DRC EUs chose cassava root flour from the other mixtures as the most practical and viable on all metrics.

**Fig 2:**
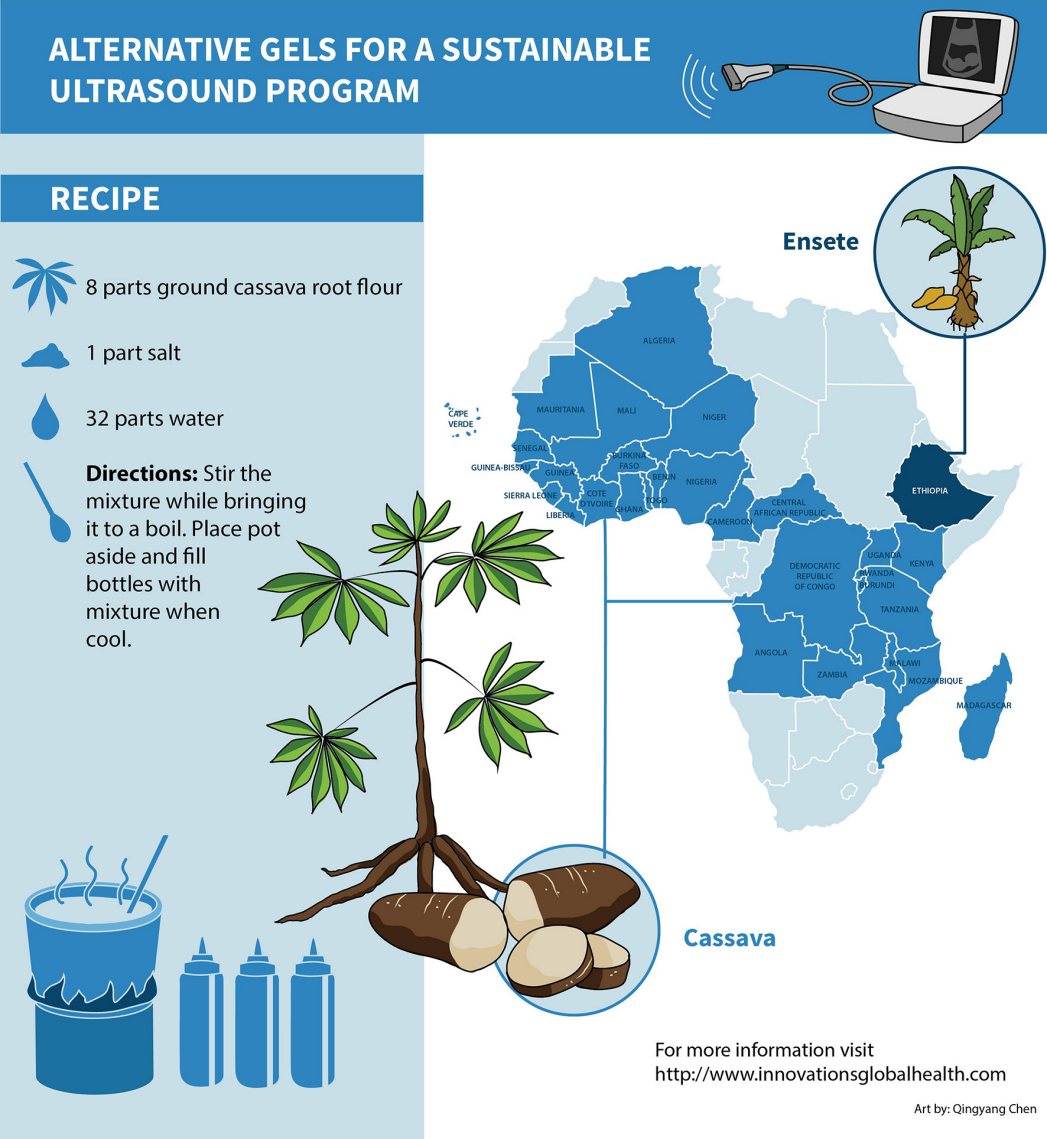
Map of gel availability on African Continent. Map of African Continent with geographic distribution of cassava (light blue) and bulla (dark blue). Sorghum is available in the pale blue states.

Six different prototypes of cassava-root were developed by heating varying concentrations of ground cassava root, salt and water, brought to a low boil while mixing. Samples were allowed to cool for 2 hours before testing. End-Users chose the mixture of 1 part salt, 8 parts cassava root flour and 32 parts water for the preferred mixture. Stir the mixture while bring to boil. Place pot aside and when cool fill bottles by spooning mixture. Ethiopian End-Users chose bulla, a flower derived from Enset (banana like plant) via a similar process. (Fig 2)

**Fig 3:**
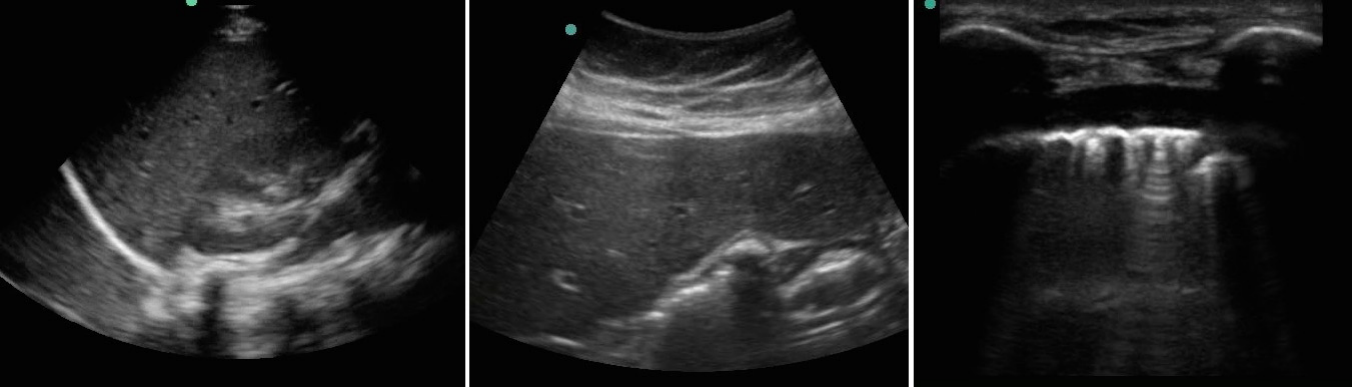
Ultrasound images taken with cassava gel. Images of the right upper quadrant FAST exam, positive gall stones and lung sliding taken with a Sonosite 180 ultrasound machine using cassava gel prototype.

Image evaluation via eight paired comparison images taken with prototype and commercial for comparison showed no preference between images from three independent evaluators. Views were typical hepato-renal FAST images, right upper quadrant, median nerve block images and lung sliding video. There were no skin reactions to gel prototypes reported. (Fig 3).

Per unit cost was calculated using the average price as quoted by market vendors in each market. Capital costs of the heat source, specifically an electric hot plate were not included. Local cost of an electric hot plate (stove) averages $50 (USD). While seemingly inexpensive, this belies the reality of the situation on the ground, in that the local health infrastructure is in such disrepair that even this cost might be considered ‘out of reach’. Therefore, the manufacture process was done using a simple charcoal heat source. Labor costs were also not included as radiology staff manufactured the prototype during the study period and continue to do so to date. The cost was absorbed into their existing salary. Cost per bottle (one days use) was calculated using 600 Congolese Francs per $1.00 and 20.1 Birr per $1.00. (Table 1)

**Table 1:** Marginal costs of cassava and bula gel.

	Cassava (1 kg bag)	Bula (1 kg bag)
Cost (ground root)	0.75USD	3.00USD
Salt (250 gm)	0.50USC	0.50USD
Bottles 250 ml (recycled)	negligible	Negligible
Number of 250ml bottles	14	14
**Total Cost per bottle**	**0.09USD**	**0.25USD**

Marginal costs of cassava are 0.08USC and bula is 0.25USD per 240ml bottle. This is assuming no waste and perfect efficiency in production.

## Discussion

Ultrasonography is one of the most important diagnostic tools for clinicians in low-resource settings. Increasingly higher resolution machines are being developed at lower costs and in portable sizes, and can be found many areas traditionally termed low-resourced. However, limited supply and recurring cost of consumables required to operate the equipment, such as ultrasound gel, is a barrier to sustainable use.

Research presented here describes the application of HCD process to generate gel alternatives from readily available low-cost ingredients in local markets that required minimal processing resources in time or energy to manufacture. We created prototypes from cassava flour (widely available through the Great Lakes Region and Western Africa), and bula in Ethiopia (most widely used food staple in Ethiopia). [13]

Both cassava and bula manufactured gel are practical alternatives from a cost perspective. The cost of commercial gel is $5.00 to >$15.00 per bottle and user groups often do limited or compromised quality scans to conserve its use. The cost of production for alternative gel in DRC was approximately ~$0.09 (USA) per 500 ml bottle. For hospitals with limited budgets, access to cheap gel could literally help sustain an ultrasound program. Indeed, Kindu General Hospital, a site with the most difficulties, now uses the cassava-root based gel for all its clinic and hospital ultrasound needs. The alternative had been no gel.

As multiple countries on the African Continent are considered low-income countries, we chose to map a broad geographic range to give health care providers alternatives. While only Ethiopia has bula (unique growing conditions), cassava appears to be ubiquitous and is reported in over 40 countries. As well, the countries not known to have cassava as a staple do have sorghum in common. One could extrapolate that sorghum could similarly be used to manufacture gel.

We found HCD methodology helpful in this setting as our user group had the opportunity to clearly state their problem contextualized to their needs and then provide feedback throughout the design process. This kept our design process on a path to a user-centered solution. A multidisciplinary team was also essential as HCD is specifically engineered such that different perspectives keep the team thinking outside the box and contribute multiple perspectives and funds of knowledge to the discussion.

Future research will extend in-field testing to include a more intensive review of image quality, as well as considerations of potential bacterial load and effects on bacterial load of in-field processing options (such as boiling time and ingredients), and potential for allergic reaction or skin irritation. The Gynecology and Radiology Department of Albert Einstein School of Medicine are currently completing these studies) As previous publications noted, starch like gel alternatives such as cornstarch show excellent ultrasound imaging quality however this raw material is not universally available. Ultimately, we hope to source local knowledge to create an interactive map of the African continent where clinicians can link to manufacture instructions as we have done in Fig 3 based on the region of interest but also to include validity testing results.

## Limitations

Neither bacterial, skin irritation / allergic reacting, nor image quality testing was conducted with complete scientific rigor. The alternative gels outlined here are intended as a pragmatic solution to a chronic resource problem witnessed in-field. All the processes outlined above will be repeated in laboratory conditions, with guidance documents released with tested and validated results.

Repeatability is a question due to the potential for inconsistent condition of the raw material. Using Cassava Root as an example; the process is feasible because finely ground cassava root is ubiquitously available across central and eastern DRC in flour form at local markets for a low cost. Consistency of the grinding process is, however, a question and wholly dependent on local conditions. Thus, the mixture identified as preferred in this paper might have to be adjusted based on locally available supplies.

The ultrasound image quality was judged qualitatively with loose criteria and there is risk of poor interobserver variability. The cost metric of 1/100^th^ the value of commercial gel was an arbitrary selection. It is quite possible that the final costs calculated will continue to be a barrier to utility.

## Conclusion

Using HCD methodology, we determined that with exceedingly low cost and ubiquitous availability of both cassava root flour in the Great Lakes region of Sun-Saharan Africa and bulla in Ethiopia there are possible low cost attractive alternatives to commercial gel. Preliminary tests show each to be equal in image quality to currently available commercial gel.

## Supporting Information

S1 Report Report from Mali, Addis Ababa and Goma. Reports by research assistants given to the Design Team after market interviews show vendors providing samples of materials that could be made into a gel slurry. Vendors in Mali manufactured a cassava slurry for the assistants to demonstrate the slurry viscosity. Suggestions in Democratic Republic of Congo were listed on notebook paper.
(PDF)

S1 Video Market women explaining the properties of cassava to research assistant in DRC. (ZIP)
