# Correction: Alternative Ultrasound Gel for a Sustainable Ultrasound Program: Application of Human Centered Design

**DOI:** 10.1371/journal.pone.0138400

**Published:** 2015-09-14

**Authors:** The PLOS ONE Staff

The seventh author’s name is spelled incorrectly. The correct name is: Sarah K. Wendel. The publisher apologizes for the error.
