# Supplementary material for: Alternative Ultrasound Gel for a Sustainable Ultrasound Program: Application of Human Centered Design
Source: PLoS One. 2015 Aug 7;10(8):e0134332. doi: 10.1371/journal.pone.0134332 (PMC4529075; doi:10.1371/journal.pone.0134332)
Supplement: S1 Report — Reports by research assistants given to the Design Team after market interviews show vendors providing samples of materials that could be made into a gel slurry. Vendors in Mali manufactured a cassava slurry for the assistants to demonstrate the slurry viscosity. Suggestions in Democratic Republic of Congo were listed on notebook paper. (PDF) [file pone.0134332.s001.pdf]

Etude sur le gel servant lors des échographies

Bameko, Mali

Après démonstration et explication sur le gel, j'ai rencontré un premier temps un groupe de femmes(04)dans le quartier de Sébénicoro qui fabriquent des savons durs et liquides chez elle pour ensuite les vendre aux grossistes qui vendront également à leur tour en détail dans les marchés locaux. Ces femmes après tâtonnement dans les mains ont suggéré au manioc pour l'approche de la couleur du gel pour Ultrasons thérapeutique. Ensuite aux fruits d'un arbre qui viennent du Mali qu'on appelle Mali Yirini dont les fruits mûrs ont presque la consistance du gel qu'elles viennent de tester…ci-dessous les photos...

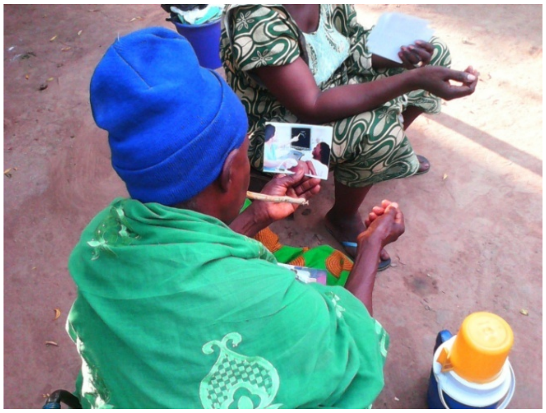

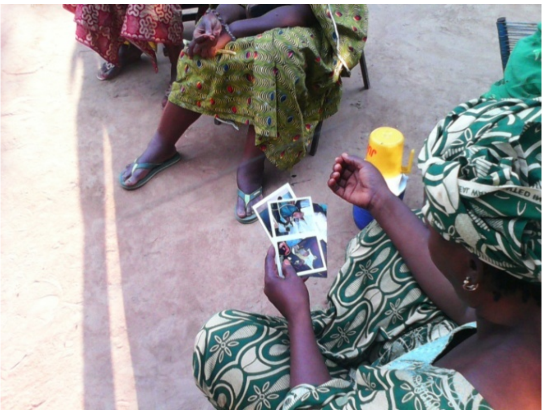

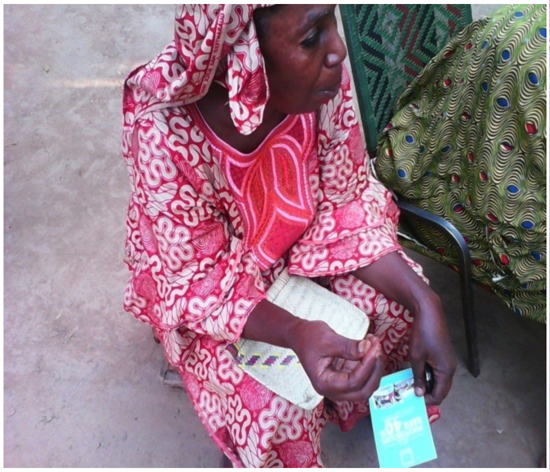

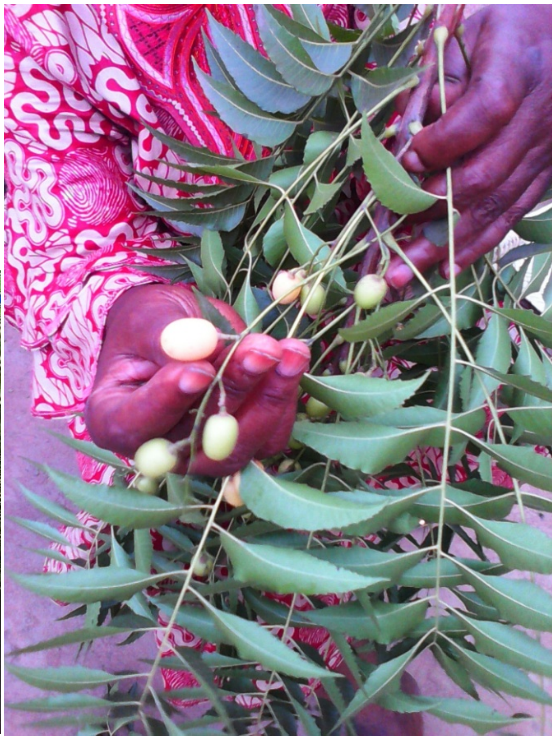

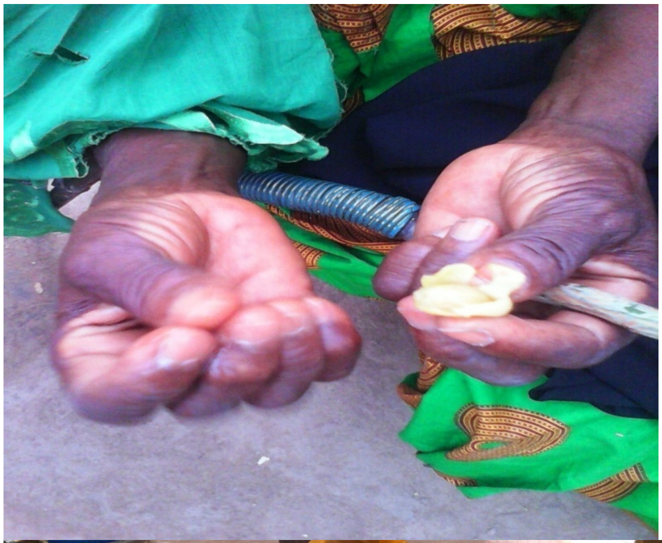
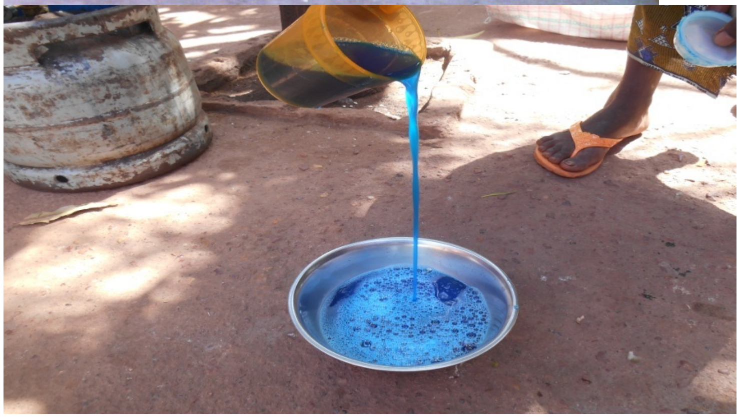
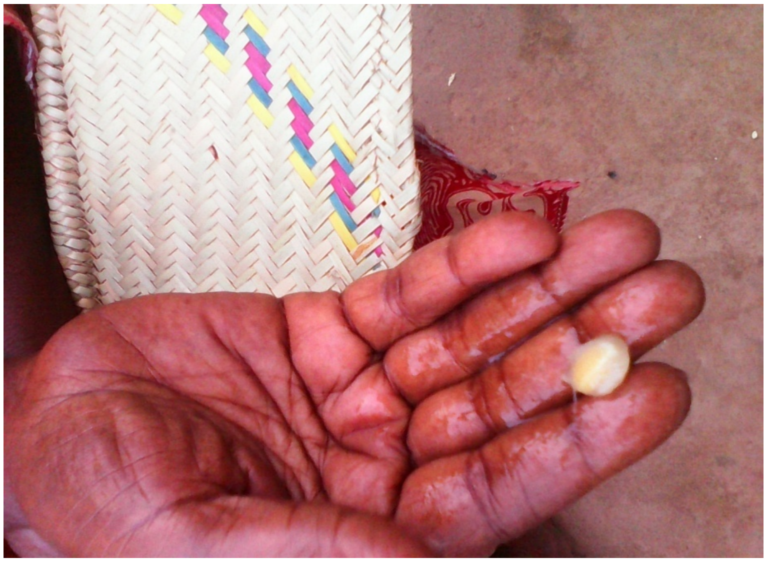

Le lendemain, je me rendis au marché d'un autre quartier (le marché de Kalaban coura) pour rencontrer d'autres femmes vendeuses de farine de manioc. Chez nous les femmes vendeuses de farines de manioc sont différentes car certains produits très différents de farine ne sont

généralement trouvé qu’avec elles tel les savons locaux, les éponges végétales pour faire la douche, du henné que les femmes utilises pour se noircir les pieds ou les mains, des bijoux en cuivre, des lianes pour parfumer l’eau à boire etc.…

Une première femme confirme la farine de manioc pour non seulement l’approche de la couleur mais aussi de l’odeur tout en précisant que c’est produit qu’on peut trouver sur place et qui est tout aussi moins cher…ci-dessous la photo

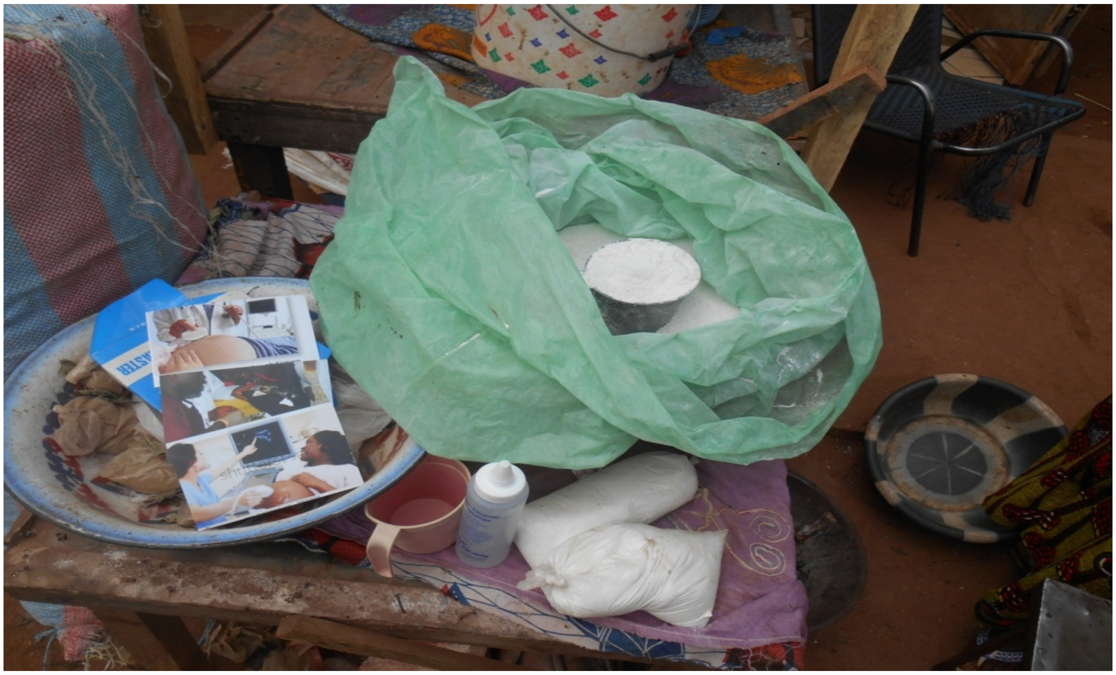

Cette deuxième femme confirme également pour la farine du manioc mais a suggéré une autre substance que j’ignore le nom en français mais qui s’appelle Yiri gnèdji en bambara ce qui signifie les larmes de l’arbre sinon l’ambre en français…c’est une substance liquide (de couleurs multiples dépendant du type d’arbre) qui s’écoule de certains arbres quand on y fait des blessures assez profonde. Au moment de l’écoulement du liquide de l’arbre c’est à l’état gel mais plus ça reste en contact avec l’air ça se durcisse comme de la pierre. A l’état pierre c’est plus conservable et en cas de besoin qu’il suffit de les réduire à l’état de farine et ajouter de l’eau chaude 24h avant utilisation…ci-dessous l’image…

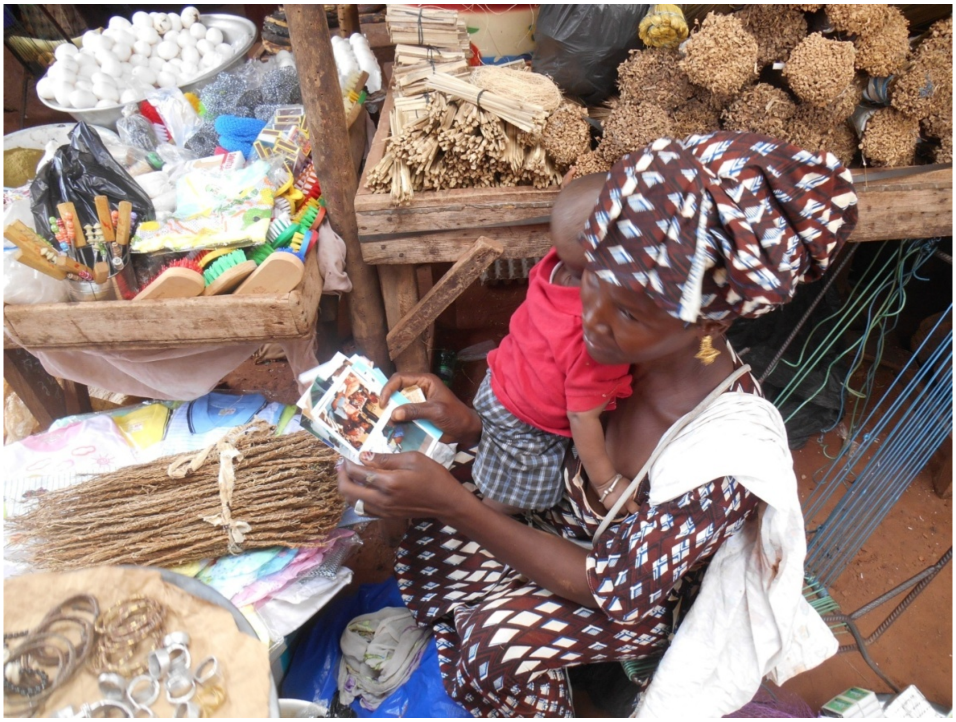

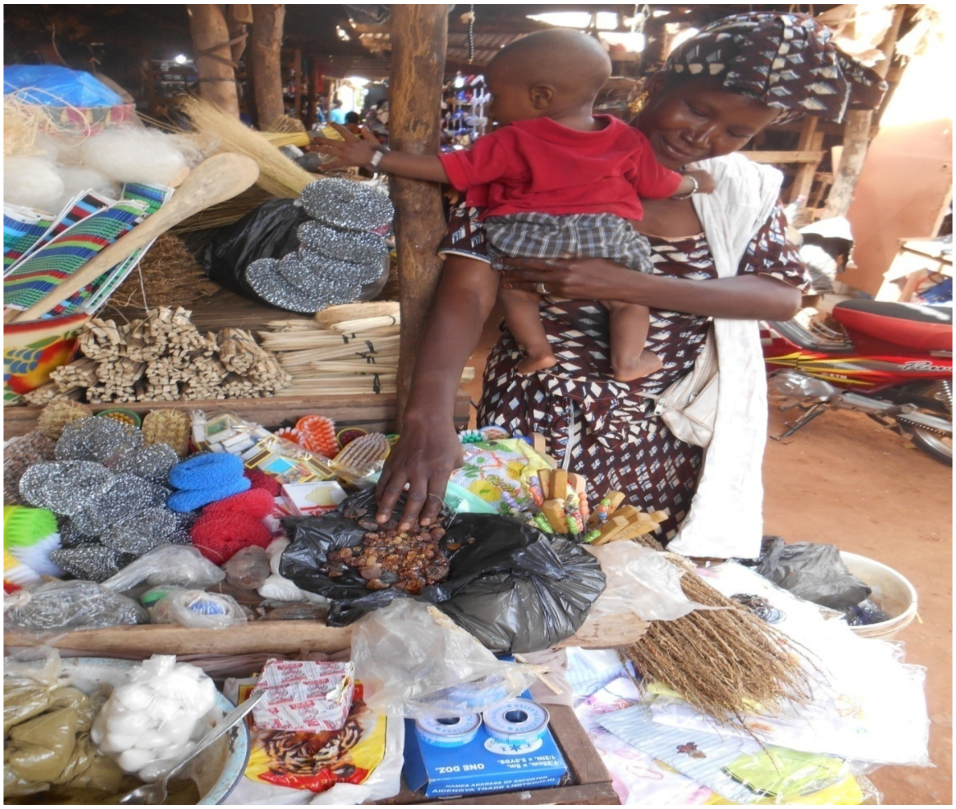

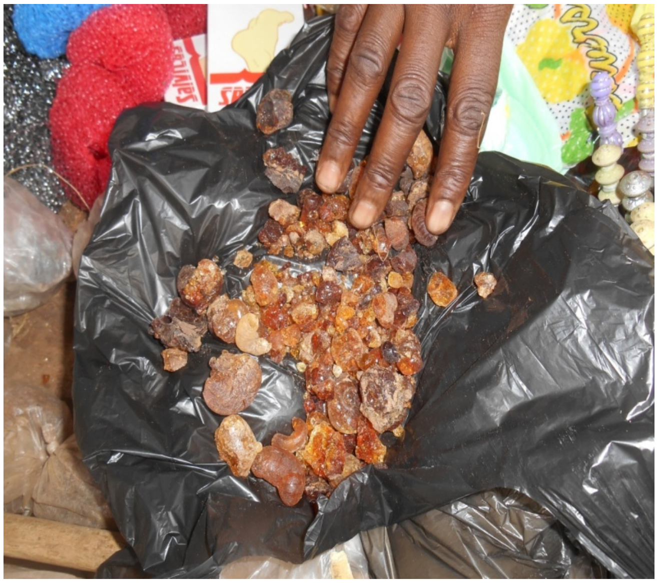

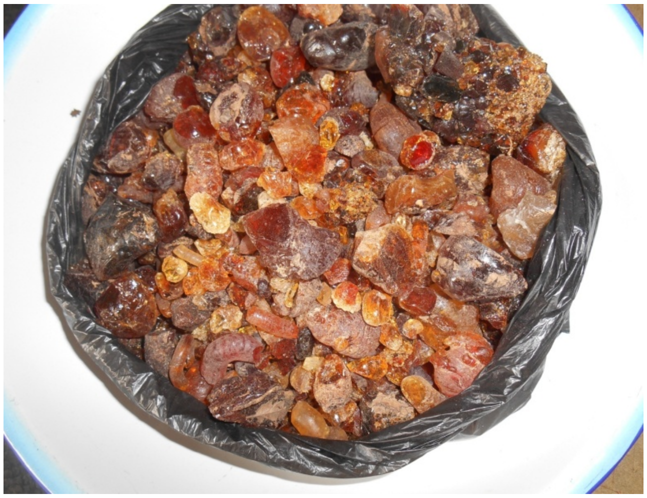

Cette autre vendeuse Dans le marché de Niamakoro propose la farine de manioc ….image ci-dessous

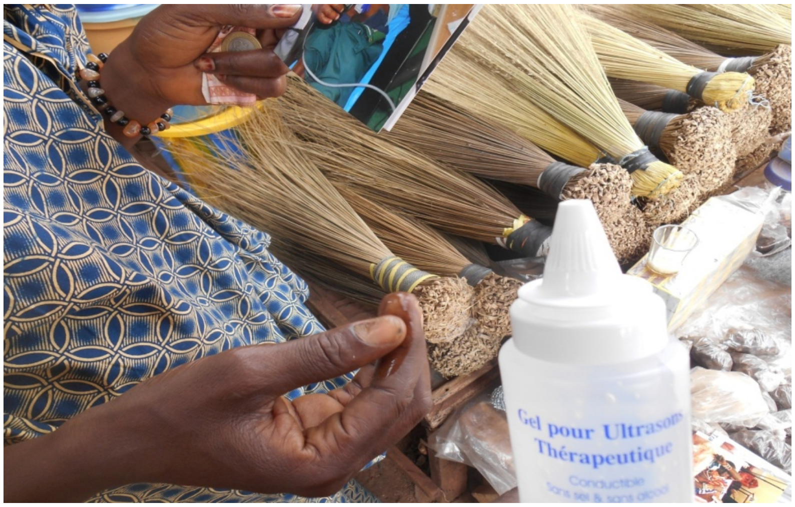

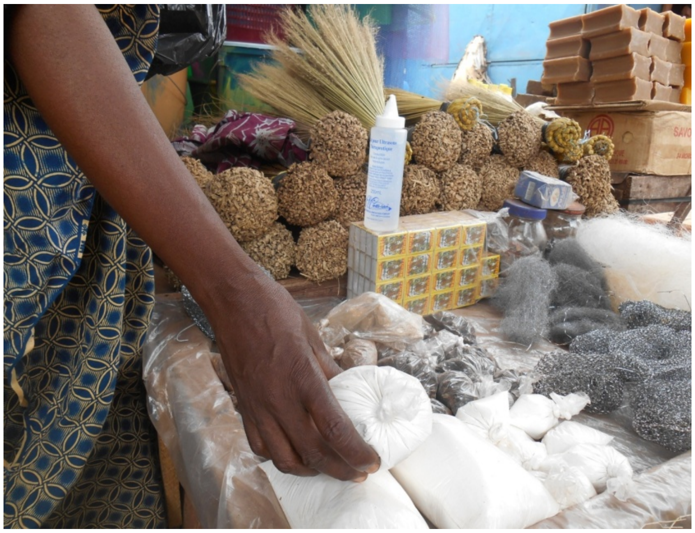

Trois autres dans le même marché de Niamakoro proposent la farine de manioc…

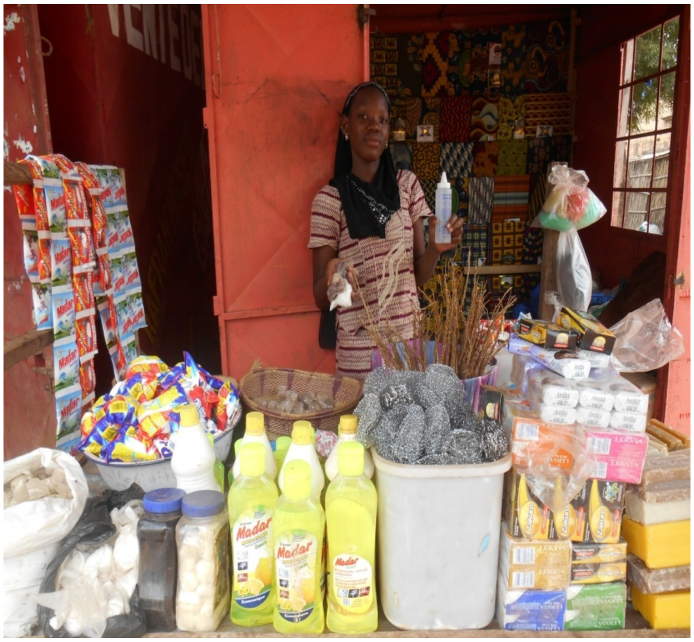

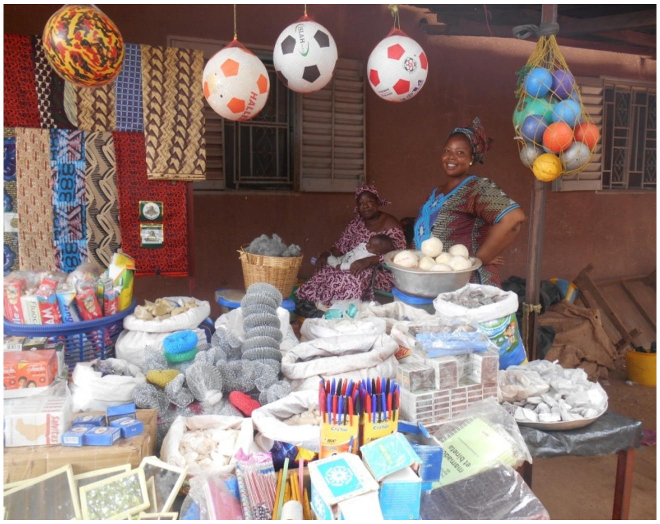

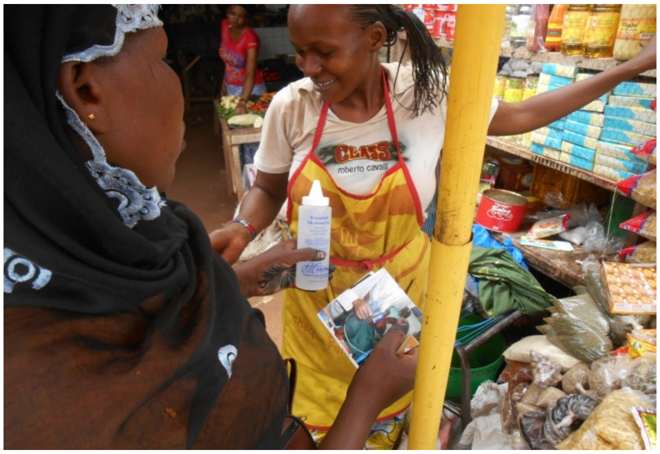

Celle la est une vendeuse d'œufs(au marché de Banankabougou) enceinte de 6 mois et dit avoir touché ce gel une fois lors de son échographie…Elle pense que le blanc d'œuf très gluant peut remplacer ce gel …image ci-dessous

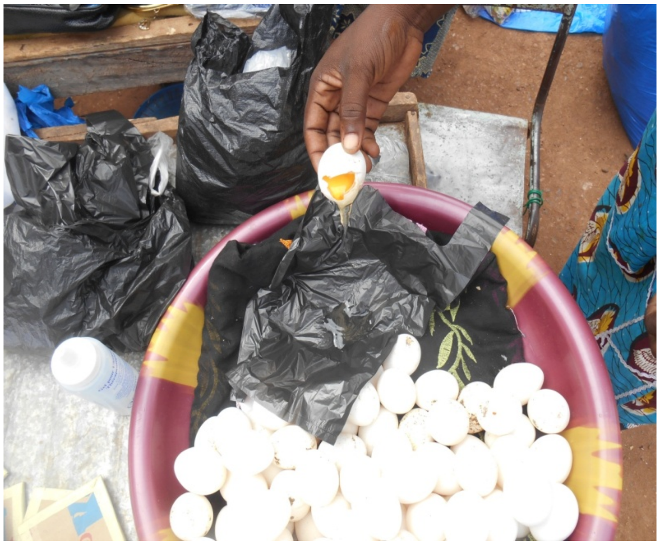

Au marché de Torokorobougou cette autre femme vendeuse de farine proposa toute de suite la farine de manioc et m'a invité à voir la préparation que vous verrez dans les photos ci-dessous …

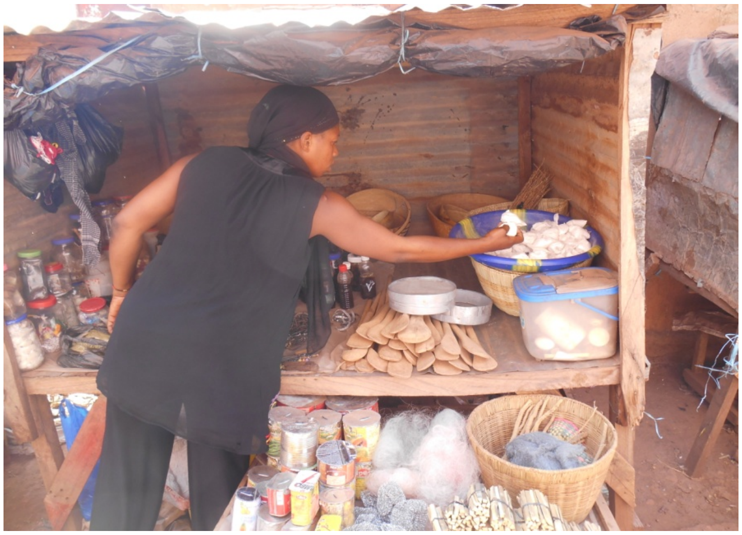

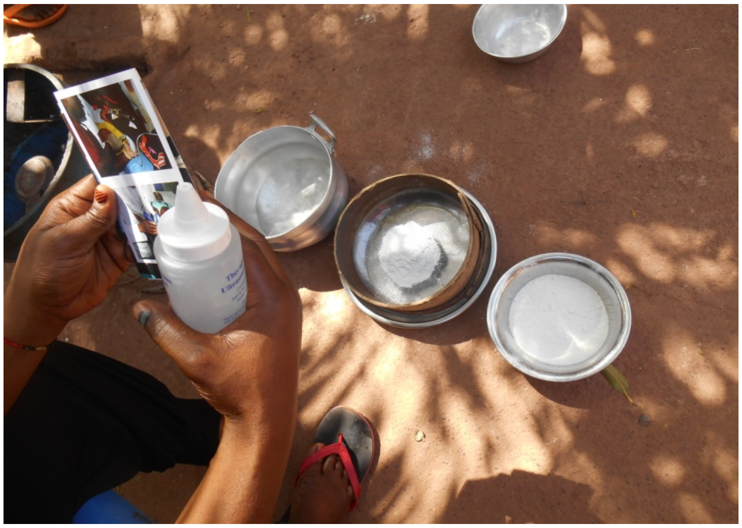

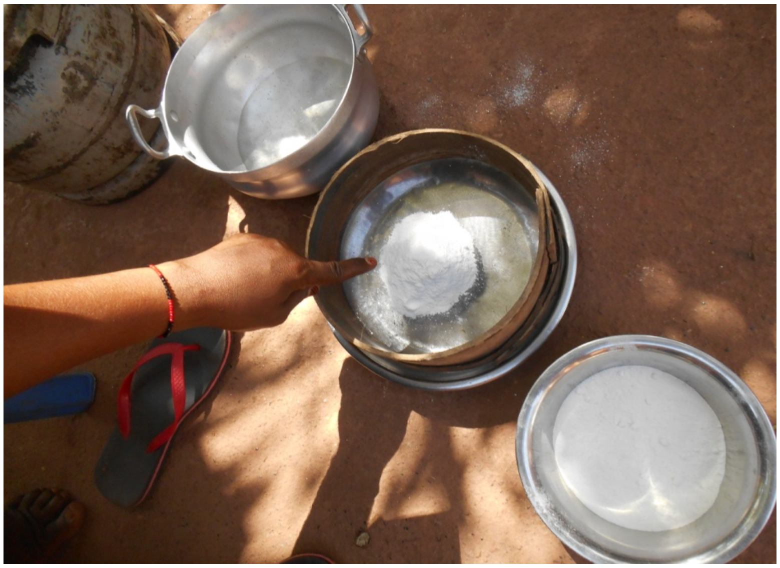

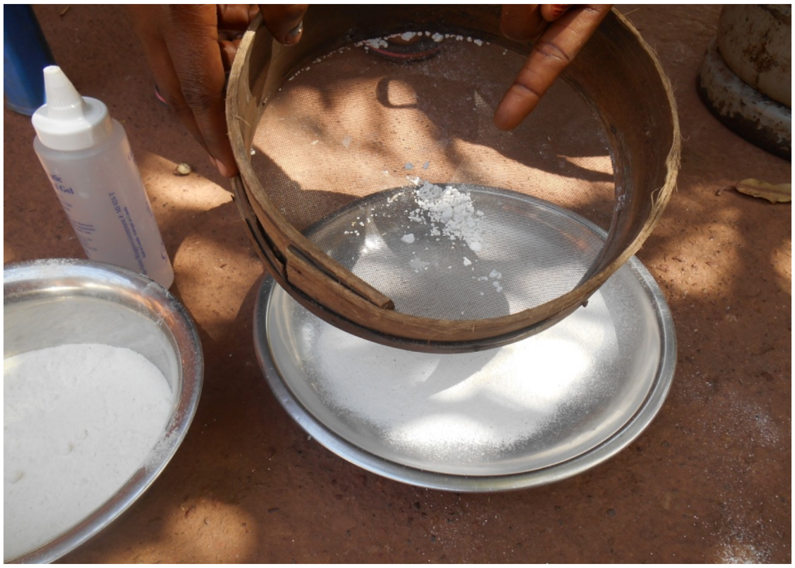

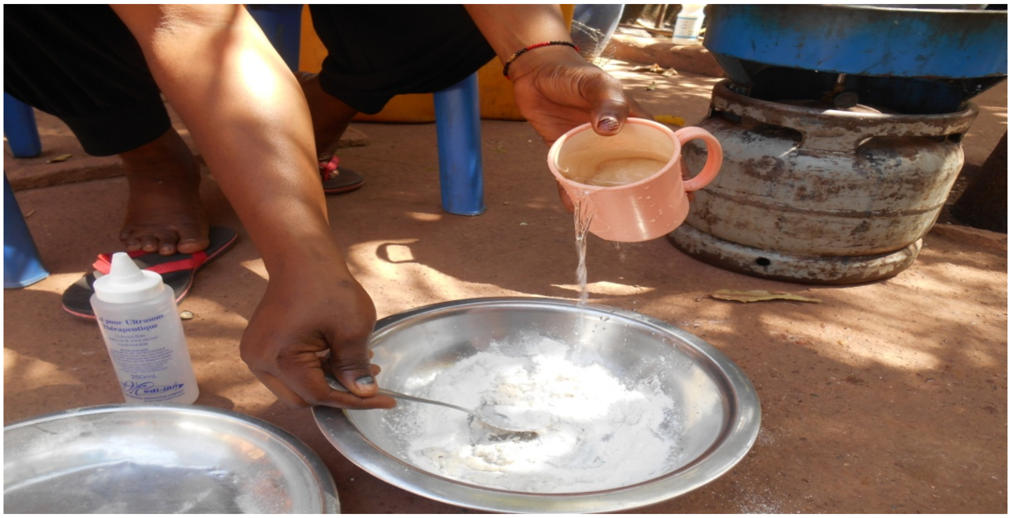

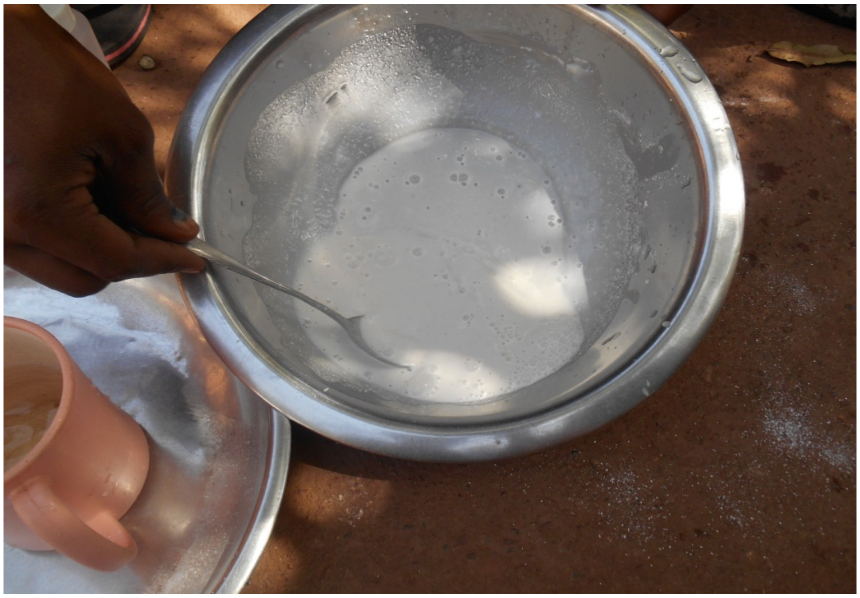

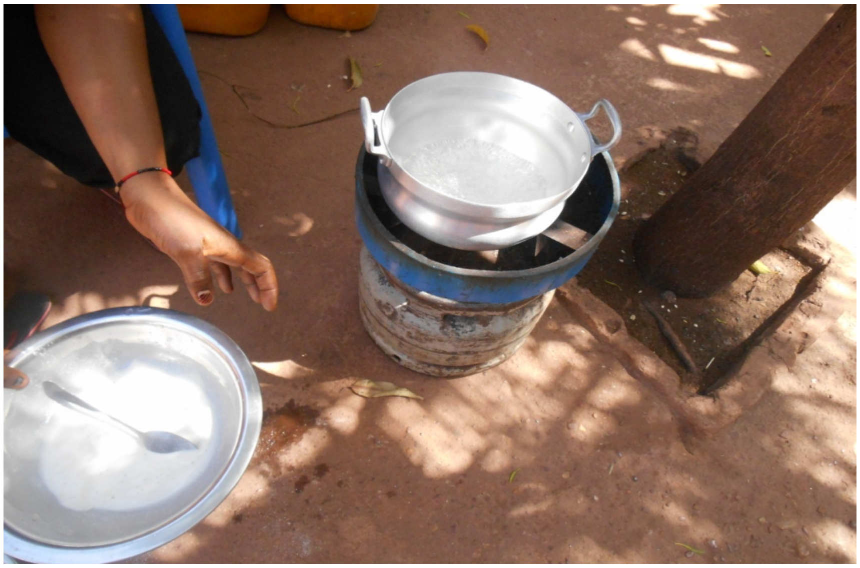

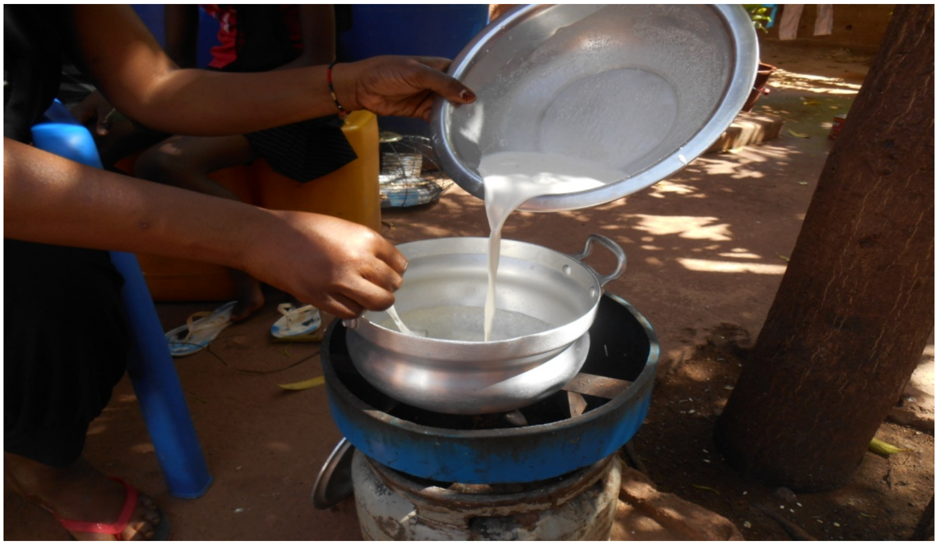

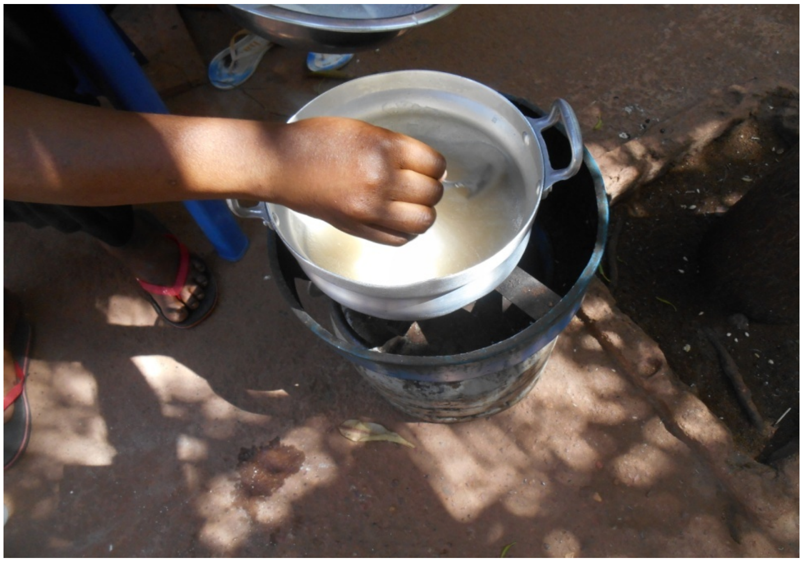

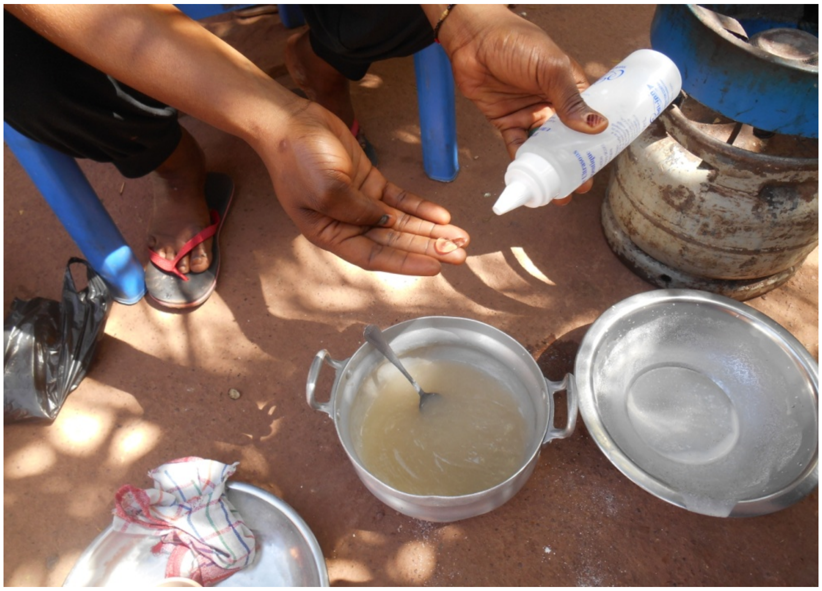

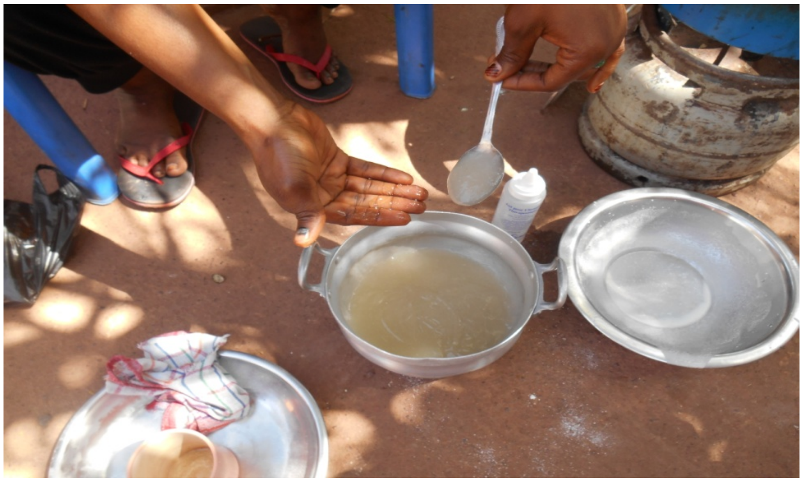

NB : Le produit plus souvent suggéré est la farine de manioc est le plus généralement élaborée par les teinturières qui l'utilisent ce gel pour la consistance d'une étoffe de tissu appelée BAZIN qui est beaucoup sollicité par les femmes lors des cérémonies de mariage, baptême etc…La farine de manioc appelée Banankou moukou dans la langue locale qui s'appelle le Bambara(Banakou=manioc et moukou=farine) est assez abondante chez nous ici au Mali malgré qu'on se fait fournir par la Côte d'Ivoire qui est un pays grand tropical producteur du manioc dans la sous région. La farine est vendue par mesure et le prix varie selon les marchés, mais toujours accessible.

Tout au long de l'enquête dans le quartier de Sébénikoro avec ce groupe de femmes qui élaborent les savons soit durs ou liquides et dans les différents marchés (Kalaban Coura,Niamakoro et Banankabougou) c'est la langue bambara qui était le moyen de communication.

Market Merkato, Addis Ababa, Ethiopia

Suggestions are:

Selit flour (sesame), telba, bula (flour from ensset), ajji, gondare and ret

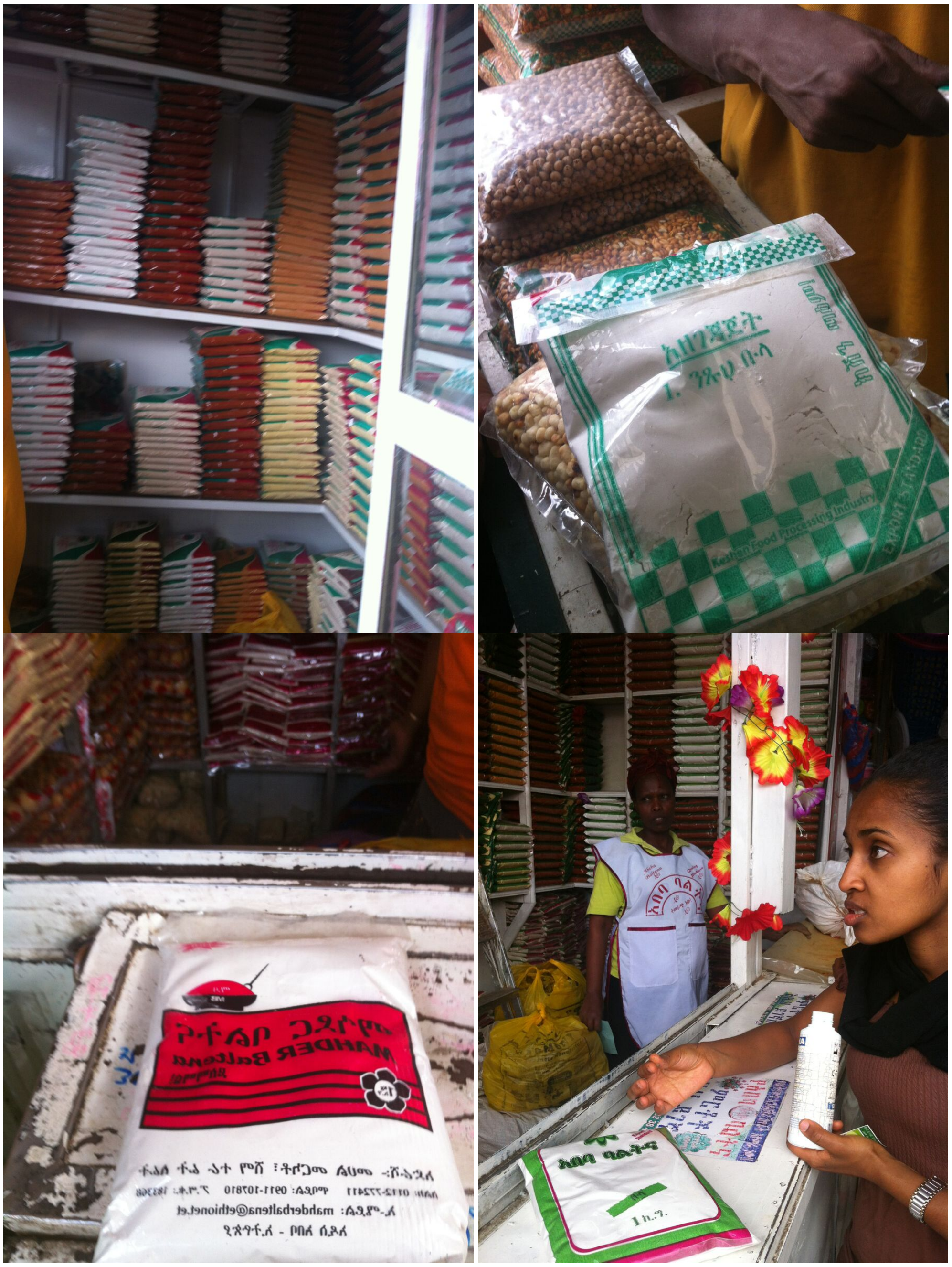

Democratic Republic Congo

Cassava

Aloevera

hand sanitizer

eggs

mix eggs | lemons | cassava

Sorgum

Plantains
